# A novel biomarker panel for irritable bowel syndrome and the application in the general population

**DOI:** 10.1038/srep26420

**Published:** 2016-06-06

**Authors:** Zlatan Mujagic, Ettje F. Tigchelaar, Alexandra Zhernakova, Thomas Ludwig, Javier Ramiro-Garcia, Agnieszka Baranska, Morris A. Swertz, Ad A. M. Masclee, Cisca Wijmenga, Frederik J. van Schooten, Agnieszka Smolinska, Daisy M. A. E. Jonkers

**Affiliations:** 1 Top Institute Food and Nutrition (TIFN), Wageningen, The Netherlands; 2 Division Gastroenterology-Hepatology, Department of Internal Medicine, NUTRIM School for Nutrition, and Translational Research in Metabolism, Maastricht University Medical Center+, Maastricht, The Netherlands; 3 Department of Genetics, University of Groningen, University Medical Centre Groningen, Groningen, The Netherlands; 4 Department Developmental Physiology and Nutrition, Danone Nutricia Research, Utrecht, The Netherlands; 5 Laboratory of Microbiology, Wageningen University, Wageningen, The Netherlands; 6 Department of Pharmacology and Toxicology, NUTRIM School for Nutrition, and Translational Research in Metabolism, Maastricht University Medical Centre+, Maastricht, The Netherlands

## Abstract

Biological markers that measure gut health and diagnose functional gastro-intestinal (GI) disorders, such as irritable bowel syndrome (IBS), are lacking. The objective was to identify and validate a biomarker panel associated with the pathophysiology of IBS that discriminates IBS from healthy controls (HC), and correlates with GI symptom severity. In a case-control design, various plasma and fecal markers were measured in a cohort of 196 clinical IBS patients and 160 HC without GI symptoms. A combination of biomarkers, which best discriminates between IBS and HC was identified and validated in an independent internal validation set and by permutation testing. The correlation between the biomarker panel and GI symptom severity was tested in IBS patients and in a general population cohort of 958 subjects. A set of 8 biomarker panel was identified to discriminate IBS from HC with high sensitivity (88.1%) and specificity (86.5%). The results for the IBS subtypes were comparable. Moreover, a moderate correlation was found between the biomarker panel and GI symptom scores in the IBS (*r* = 0.59, *p* < 0.001) and the general population cohorts (*r* = 0.51, *p* = 0.003). A novel multi-domain biomarker panel has been identified and validated, which correlated moderately to GI symptom severity in IBS and general population subjects.

Interest in ‘gut health’ is rapidly emerging^1^. Gastrointestinal (GI) symptoms and disorders occur frequently in the general population and may reflect compromised gut health^2^. Although gut health is difficult to define, five major domains related to a healthy GI system have been proposed by Bischoff. These domains include effective digestion and absorption, normal and stable microbiota, effective immune status, absence of GI illness and status of general well-being^1^. As various underlying mechanisms may affect these domains, it is challenging to identify and validate markers for gut health. This is particularly relevant when healthy subjects are compared with subjects with functional GI disorders, or when evaluating potential benefits of dietary interventions in the general population. For organic GI diseases, diagnostic tools and markers are generally available in daily clinical practice, such as GI endoscopy, histological examination of tissue samples or biomarkers (*e.g*. anti-transglutaminase antibodies)^3^,^4^. However, objective biological markers that aid the diagnostic process and facilitate follow up and assessment of treatment efficacy of functional GI disorders, such as Irritable Bowel Syndrome (IBS), are lacking. Neither are markers available that correlate well with GI symptom severity^5^,^6^.

The Rome III symptom criteria are the current gold standard for the diagnosis of IBS. Although the Rome committee emphasizes that IBS is not a diagnosis of exclusion and should be based on symptom criteria, in daily clinical practice endoscopy, CT or ultrasound imaging, blood, fecal or other analyses are frequently used to exclude organic disorders^7^. Therefore, there is increasing interest in reliable biomarkers that can aid the diagnosis of IBS^8^,^9^. A clinically and scientifically relevant biomarker for IBS should be able to a) discriminate IBS patients from healthy subjects, b) discriminate IBS patients from subjects with organic GI disorders, and c) correlate with severity of GI symptoms, to be useful for monitoring of treatment efficacy. Finding objective biomarkers that correlate with the severity of GI symptoms in IBS is also an important step towards suitable biomarkers for functional GI complaints and gut health in the general population.

Up to now, use of non-invasive single biomarkers for IBS has led to only moderate results^5^,^6^. Therefore, recent studies focused on biomarker panels^9^. To date, a specificity and sensitivity of 88% and 50%, respectively, has been reported for a 10 item-serum marker panel^10^, and of 64% and 81%, respectively, for a 34 item-serum/genetic marker panel for the discrimination of IBS patients versus healthy controls^11^. Though promising, biomarkers with higher predictive values are needed for daily clinical practice and scientific research. We postulated that combining markers that reflect the multifactorial pathophysiology of IBS may lead to better results. Strong evidence exists for a combination of rather subtle pathophysiological changes, such as low-grade mucosal and systemic immune activation, intestinal barrier dysfunction, dysregulation in neural and neuro-hormonal signaling and altered microbiota and host-microbe interaction, that contribute to symptom generation in IBS^12^. These mechanisms do overlap with the domains of gut health as described by Bischoff^1^.

In the current study, we hypothesized that biomarkers related to several domains of gut health and associated with the pathophysiology of IBS, can be combined and used as a highly sensitive and specific biomarker panel to discriminate clinically diagnosed IBS patients form healthy controls. Secondly, we hypothesized that this biomarker panel correlates with GI symptom severity, not only in IBS patients derived from a clinical setting with relatively high symptom burden, but also in subjects with mild to moderate GI symptoms from the general population.

The primary objective of the present study was to assess levels of selected plasma and fecal biomarkers, related to several domains of gut health, and to identify and validate a non-invasive biomarker panel, which best discriminates IBS patients from healthy controls. The secondary objective was to correlate the biomarker panel to GI symptom severity in clinical IBS patients as well as in a large general population cohort.

## Methods

In a case control design, two large Dutch cohorts were used, *i.e*. a clinical cohort of IBS patients and healthy controls without GI symptoms (Maastricht IBS (MIBS) cohort)^4^,^13^, and a general population cohort (LifeLines (LL) DEEP)^14^. From both cohorts, subjects were included in the analyses if fecal and plasma samples and completed GI symptom scores were available. Identification and validation of the biomarker panel was conducted in clinical IBS patients and healthy controls of the MIBS cohort. The correlation of the biomarker panel to GI symptoms was evaluated in both cohorts.

### Study participants; MIBS cohort

IBS patients, between 18 and 75 years of age, diagnosed by their treating physician and fulfilling Rome III criteria, were recruited via the outpatient gastroenterology-hepatology clinic of MUMC+, a secondary and tertiary referral center, and via general practitioners in the area of Maastricht. Medical history was taken and GI endoscopy with biopsies, abdominal imaging and/or blood, breath and fecal analyses were performed to exclude organic disease, when indicated. Patients with a history of abdominal surgery, apart from appendectomy, laparoscopic cholecystectomy and hysterectomy, were excluded. Patients were assigned to IBS subtypes based on predominant bowel habits according to the Rome III criteria: diarrhea (IBS-D), constipation (IBS-C), mixed stool pattern (IBS-M) and unspecified subtype (IBS-U). Age and sex matched healthy controls (HC) were enrolled via public advertising. A brief medical history was taken to exclude the presence of previous or current GI disorders or complaints. All study participants completed questionnaires regarding demographic characteristics and lifestyle factors. A 14-day end-of-day GI symptom diary was completed, addressing symptoms of abdominal discomfort and pain, nausea, bloating, belching, flatulence, diarrhea and constipation, on a 1-to-5 point Likert scale^4^.

### Study participants; LL DEEP cohort

Study participants above 18 years of age and regardless of their medical background or current complaints were randomly enrolled from a large general population cohort in the Netherlands, *i.e*. LifeLines^15^. In a subgroup of the cohort, referred to as LifeLines Detailed Extensive Examination of Participants (LL DEEP), among others fecal and plasma samples were available and subjects completed self-report questionnaires on demographics and lifestyle factors, as well as a 7-day GI symptom diary, which was identical to the 14-day diary used in the MIBS cohort^14^. Information on clinical diagnoses of IBS was not available for this general population cohort.

All study participants gave written informed consent prior to inclusion. Study protocols were approved by the Maastricht University Medical Center+ (MUMC+) and University Medical Centre Groningen (UMCG) Ethics Committees, respectively, and were in compliance with the revised Declaration of Helsinki (64th WMA General Assembly, Fortaleza, Brazil, 2013). The MIBS cohort was registered in the US National Library of Medicine (http://www.clinicaltrials.gov, *NCT00775060*).

### Selection of biomarkers

Forty-three potential biomarkers were selected based on extensive literature search, using the following selection criteria: i) markers are related to one or more gut health domains as described by Bischoff^1^; ii) they potentially discriminate between subjects with healthy gut function and IBS patients; and iii) can be measured in blood or fecal samples collected according to generally accepted and applicable procedures for bio sample collection. Based on expert panels, focusing on relevance and feasibility of the markers, fifteen biomarkers were selected to be measured (in both cohorts). Plasma citrulline, a marker of functional enterocyte mass of the small bowel, was selected as indicator of the absorptive^16^,^17^ and barrier function of the small intestine^18^,^19^. Non-stimulated plasma cytokine levels, *i.e*. IL-1β, IL-6, IL-8, IL-10, IL-12p70 and TNF-α, were selected as markers of systemic immune activation^20^,^21^. Fecal calprotectin, a calcium and zinc-binding protein, was selected as an indicator of intestinal inflammation, proportional to neutrophil migration towards the intestinal tract^22^,^23^. Fecal human *β*-defensin 2 (HBD2), a human antimicrobial peptide produced by among others intestinal epithelial cells in response to (pathogenic) bacteria, was selected as an indicator of host defense against microbes^24^,^25^. Fecal short chain fatty acids (SCFAs), *i.e*. acetate, propionate, butyrate, valerate, and caproate, are products of microbial fermentation of non-digested oligosaccharides in the colon, and were selected as indicators of gut intraluminal metabolic activity. SCFAs have been associated to multiple pathological and physiological mechanisms in humans, *e.g*. modulation of inflammation, satiety, carcinogenesis, and are an important energy source for colonocytes^26^,^27^. Fecal chromogranin A (CgA), was selected as an indicator of the intestinal neuroendocrine cell activity. This peptide is produced by among others enterochromaffin cells, and is co-localized in storage granules with serotonin^23^,^28^,^29^,^30^.

### Measurement of biomarkers

Fecal and blood samples were collected using standardized collection procedures. Venous blood was collected in K_2_EDTA (BD Vacutainer®) tubes and centrifuged to obtained plasma supernatants, which were aliquoted and frozen at −80 °C until analysis. In the same time period, subjects collected stool samples, which were aliquoted and stored at −80 °C within 24 hours after defecation. All markers were measured simultaneously to avoid additional thaw cycles, by ‘Medische Laboratoria Dr. Stein & Colleges’, The Netherlands. Calprotectin and HBD2 were assessed using a commercial enzyme-linked immunosorbent assay (ELISA, by Bühlmann Laboratories, Switzerland, and Immunodiagnostik AG, Germany, respectively)^23^,^25^, and CgA using a commercial radioimmunoassay (RIA, Euro-Diagnostica, Sweden)^23^, as described previously^23^,^25^. Fecal SCFAs, *i.e*. acetate, propionate, butyrate, valerate and caproate, were measured by gas chromatography-mass spectrometry (GC-MS) according to the method described by Garciá-Villalba *et al*.^31^. Concentrations of plasma citrulline were determined by high pressure liquid chromatography (HPLC) fluorescence detection, as described previously^16^. Plasma cytokines, *i.e*. IL-1β, IL-6, IL-8, IL-10, IL-12p70 and TNF-α, were measured by ProcartaPlex^TM^ multiplex immunoassay (eBioscience, USA), as described previously^32^,^33^.

### Data and statistical analysis

Levels of individual biomarkers were compared between groups using the Mann-Whitney U test, due to left skewed distributions, with post-hoc correction for multiple testing by the Benjamini–Hochberg step-up procedure^34^. The method of multiple imputations was implemented as part of the statistical procedure for biomarker levels that were below the detection limit^35^. These were randomly (with uniform distribution) replaced by levels ranging from zero till limit of detection. The assignment of values below detection limit was repeated 2000 times. All statistical results were calculated as average values over the 2000 iterations.

The data set of the MIBS cohort was then used for identification and validation of a biomarker panel that best discriminates between IBS and HC, using a supervised technique, namely Partial Least Square Discriminant Analysis (PLS-DA)^36^. This strategy for supervised data analysis via PLS-DA involved a random division of the MIBS cohort into a representative training set (80% of samples per group) and a validation set (20% of samples per group), as described by Guyon^37^. Within the training set we applied double cross-validation procedure for model optimization, the most discriminatory biomarker selection and for development of the final PLS-DA classification model^38^. The latter was done in the independent validation set of the MIBS cohort (the randomly separated 20% of samples not used in any optimization steps), by testing the performance of selected biomarker panel to classify individuals as being an IBS patient or HC.

Note, that commonly used cross-validation leads to optimistic bias (*i.e*. too low error estimate)^39^. Thus, it was not used in our analyses for evaluating the predictive power of the PLS-DA model.

Furthermore, linear correlations between the most discriminatory biomarker panel and a set of GI symptoms, including all items of the GI symptom diary were tested in the MIBS and in the general population LL DEEP cohort, using canonical correlation analysis (CCA)^40^, which is an extension of bi-variate correlation. CCA calculates a linear correlation that best explains the variability both within and between the biomarker panel and the measured GI symptoms.

## Results

### Identification and validation of the most discriminative biomarker panel for IBS versus HC

Baseline characteristics for 196 clinical IBS patients and 160 age and gender matched HC without GI symptoms (MIBS cohort) are presented in Table 1. All GI symptom scores were higher in IBS patients compared to HC. The scores for constipation and diarrhea were significantly altered, in line with the IBS subtypes (data not shown).

The biomarker data for IBS and HC subjects of the total MIBS cohort are presented in Table 2. Based on univariate comparisons, non-stimulated IL-1β and IL-6 were significantly lower, while IL-12p70 was significantly higher in plasma samples of IBS patients relative to HC. In fecal samples, CgA and calprotectin were significantly increased, while HBD2, and the SCFAs, valerate and caproate, were significantly decreased in IBS patients compared to HC. These differences remained statistically significant after correction for multiple testing.

**Figure 1:**
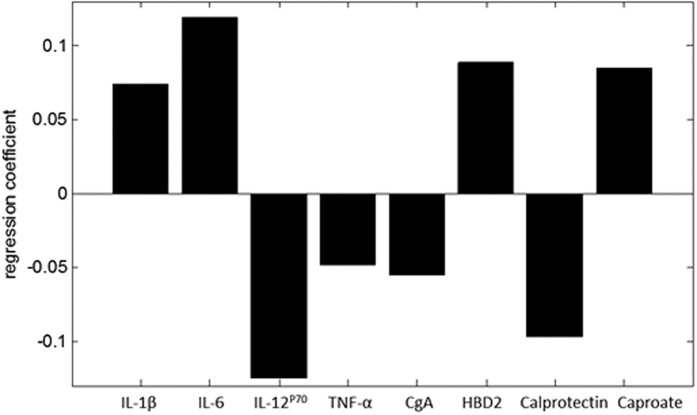
The final 8-item biomarker panel. The length of the bar, *i.e*. positive or negative regression coefficient level, indicates the relative importance of the specific marker within the biomarker panel. Positive markers are decreased in IBS patients compared to HC, and vice versa for the negative markers.

**Figure 2:**
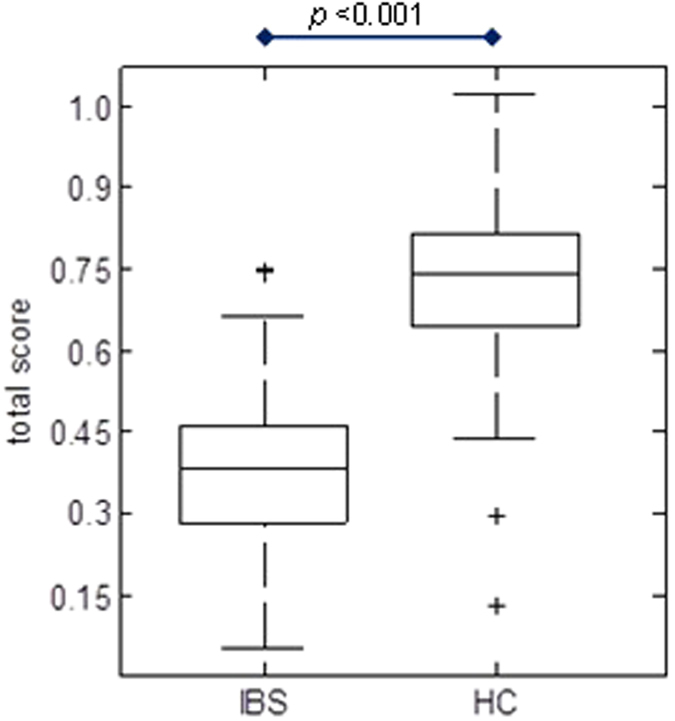
Box plot representing the total score obtained for the 8-item biomarker panel for the IBS and HC group. The total score was obtained by multiplying the real concentration of measured biomarkers in the validation set by the regression coefficient per marker (Fig. 1). It represents the probability of being IBS or HC.

For identification of the most discriminative biomarker panel, the MIBS training cohort was used. A final panel of 8 biomarkers, *i.e*. IL-1β, IL-6, IL12p70, TNF-α, CgA, HBD2, calprotectin and caproate, was identified with PLS-DA to have the best predictive value for the IBS versus HC group. This panel is presented in Fig. 1, wherein the regression coefficient level indicates the relative importance of the specific marker within the biomarker panel. Markers with a positive regression coefficient are decreased in the IBS group, and vice versa. The regression coefficients and the biomarker concentrations were used to calculate a total score for the biomarker panel per subject. This was done by multiplying the concentration of each biomarker as actually measured, by the level of importance of that specific marker within the panel obtained from the regression coefficient. The total score represents the probability of being IBS or HC. The values of these scores were found to be significantly different between the IBS versus the HC group in the validation set, as shown in Fig. 2 (p < 0.001).

**Figure 3:**
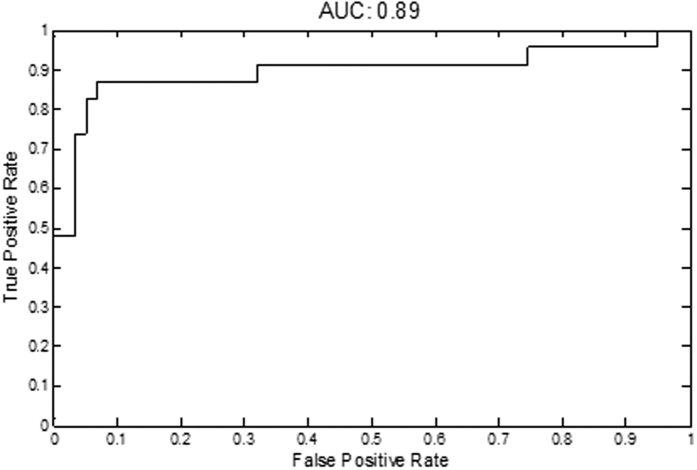
ROC curve, with an area under the curve (AUC) of 0.89 for the discrimination between IBS patients and HC in the validation set, using the final 8-item biomarker panel.

Thereafter, the pre-assigned independent validation set of IBS patients and HC of the MIBS cohort that were left out of the biomarker panel discovery step, was used to test the performance of the biomarker panel for the discrimination between IBS patients and HC. This is illustrated by the receiver operating characteristic (ROC) curve in Fig. 3, with an area under the curve (AUC) of 0.89. The line of the ROC curve represents the sensitivity and specificity of the classification model, which is derived from an algorithm representing all 8 biomarkers in the panel. The sensitivity and specificity at the optimal threshold for the diagnosis of IBS were 88.1% and 86.5%, respectively, and the positive and negative predictive values were 94.4% and 74.0%, respectively.

The results for the three largest IBS subtypes, for the set of 8 biomarkers, were comparable to the total IBS group, *i.e*. area under ROC curve of 0.86 for IBS-D, 0.80 for IBS-C and 0.89 for IBS-M in comparison to HC. These results were obtained by stratifying the number of individuals of each subtype in the validation set. Due to a small sample size in the IBS-U subtype (n = 13), the AUC was not calculated for this subtype.

In a post hoc analysis, we calculated the likelihood ratio, *i.e*. the proportion of IBS subjects with a positive test divided by the proportion of control subjects with a positive test by the biomarker panel. We are mostly interested in positive likelihood ratio since it tells the clinical concept of ruling-in disease. The negative likelihood ratio corresponds to the clinical conception of ruling-out disease. The positive likelihood ratio is equal to 6.5 and it was calculated as the ratio between the sensitivity and 100-specificity. The negative likelihood ratio of the biomarker panel is equal to 0.14 and it was calculated as the ratio between 100-sensitivity and specificity.

### Correlation between the biomarker panel and GI symptoms in the MIBS cohort

In the total MIBS cohort (*i.e*. 160 HC and 196 IBS patients), the strongest correlation, *i.e*. correlation coefficient of 0.59 (*p* < 0.001), was found between the 8-item biomarker panel (Fig. 1) and a set of GI symptoms, involving the following symptom scores: 14-day mean scores of abdominal pain, abdominal discomfort, bloating, flatulence and nausea. No correlations were found between the biomarker panel and the other GI symptoms assessed by the diary, *i.e*. belching, constipation and diarrhea.

### Application of the biomarker panel in the general population LL DEEP cohort

The potency of the biomarker set in the general population was evaluated by investigating the correlation between the 8-item biomarker panel (Fig. 1) and GI symptoms assessed by the 7-day end-of-day GI symptom diary for the LL DEEP cohort (Tables 3 and 4). Since GI symptom severity scores in the total cohort (n = 958) were low, only subjects from the LL DEEP cohort with a GI symptom diary 7-day mean score for abdominal pain and discomfort of 1.5 or higher were included in the correlation analysis. This cut-off value indicates the subject had at least 2 days with moderately-severe or 4 days with slightly-severe GI symptoms during those 7 days. The cut-of value was further supported by data of the MIBS cohort in which 95% of healthy controls had end-of-day diary mean-scores below 1.5 for these GI symptoms. A GI symptom diary mean score of 1.5 or higher was found in 221 (23%) participants of the LL DEEP cohort. In this LL DEEP subgroup, the 8-item biomarker panel was found to correlate significantly with a set of GI symptoms, *i.e*. 7-day mean scores of abdominal pain, discomfort, constipation, belching and nausea, resulting in a correlation coefficient of 0.51 (*p* = 0.003).

## Discussion

In the current study, we investigated a combination of markers related to several gut health domains and identified and validated a novel non-invasive biomarker panel, with a high predictive value (area under ROC curve of 0.89) for the discrimination between clinically diagnosed IBS patients who fulfilled the Rome III criteria and healthy controls who did not have any GI complaints. The 8-item biomarker panel correlated moderately (*r* = 0.59, *p* < 0.001) to GI symptom severity in the IBS cohort and the general population cohort (*r* = 0.51, *p* = 0.003).

In the search for a non-invasive biomarker panel for IBS, we measured 15 biomarkers in a large group of study participants. Eight markers, *i.e*. plasma IL-1β, IL-6, IL-12p70, and fecal CgA, calprotectin, HBD2, valerate, and caproate, proved to be significantly altered in IBS patients compared to HC, when tested by univariate analyses (Table 2). Of the 8 markers, 7 (valerate excluded) were part of the final 8-item biomarker panel (Fig. 1) that best discriminated IBS patients from HC. Also TNF-α was identified as one of the discriminatory markers within the final biomarker panel, while the difference between IBS and HC in the univariate analysis was not statistically significant (*p* > 0.05). This supports the advantage of a biomarker panel that includes a combination of markers, over the use of single biomarkers for the positive identification of IBS patients in a cohort that also included healthy controls.

Calprotectin and IL-12p70 were increased in IBS patients compared to HC, pointing to a low-grade pro-inflammatory state in IBS, as has been reported previously for subgroups^20^,^21^,^22^. Interestingly, IL-1β and IL-6 were decreased in IBS patients compared to HC. IBS is a chronic GI disorder, and in contrast to IL-12p70, which is as pro-inflammatory cytokine associated with chronic inflammatory conditions^41^,^42^, IL-1β and IL-6 have both pro- and anti-inflammatory properties, but are predominantly involved in the *acute*-phase immunological response^43^,^44^,^45^. This may imply that the acute immunological response in IBS patients may not be affected. It should be taken into account that we have measured baseline cytokine levels and not a stimulated cytokine response (*e.g*. by LPS). Therefore, a presumed role of the measured cytokines in the IBS pathophysiology should be interpreted with care. The level of CgA was increased in IBS patients compared to HC, as has been observed previously^23^, and may indicate an altered intestinal neuroendocrine activity in IBS patients. Finally, the antimicrobial peptide HBD2, as well as the SCFAs caproate and valerate, were decreased in IBS patients compared to HC. The interpretation with regard to IBS pathophysiology is challenging, since a decreased production as well as increased breakdown or degradation could underlie the observed differences. Nevertheless, the observed differences in fecal concentrations of these markers between IBS patients and HC are indicative of altered gut microbial activity and/or host-microbe interaction in IBS patients.

Taken together, we have observed significant but subtle differences between IBS patients and HC for several of the tested markers. Separately, in line with our hypothesis, these markers lacked potential for the discrimination between groups, but when combined had a high predictive value with a good sensitivity (88.1%) and specificity (86.5%). The results for the IBS subtypes were comparable, which indicates that the biomarker panel is useful regardless of the IBS subtype. The final 8-item biomarker panel consists of markers related to several gut-health domains, such as immunology, intraluminal processes and neuroendocrine activity.

In a recent review, Shah *et al*. tried to identify studies which evaluated IBS prevalence. Based on the meta-analysis, the authors concluded that a positive likelihood ratio of ≥5 would make an ideal biomarker for the diagnosis of IBS^46^. In our study, the positive likelihood ratio of the 8-item biomarker panel is 6.5, which indicates a moderately high probability of obtaining a positive test in the presence of IBS.

The current findings demonstrate that it is possible to identify the presence of a functional gut disorder (*i.e*. IBS) based on a non-invasive biomarker panel even with only subtle changes per single maker between health and disease status. As most clinicians (>70%) do not solely use the Rome III criteria and frequently rely on additional (invasive) investigations to make a positive diagnosis of IBS^7^, a non-invasive biomarker panel could aid the diagnostic process of IBS in clinical practice. The identification of the current ‘multi-gut health domain’ panel of fecal and plasma markers is an important step in the development of reliable biomarkers for IBS.

Certain limitations of the present study should be mentioned. We have used a clinical IBS cohort with a larger number of IBS patients relative to HC. Furthermore, in daily clinical practice there is a particular need for a non-invasive diagnostic test to distinguish between functional and organic GI disorders. Therefore, before implementation of the biomarker panel in daily clinical practice, further research is needed to assess the value of the biomarker panel for the discrimination between IBS and organic GI disorders, such as inflammatory bowel disease or coeliac disease, and its potential to monitor symptoms over time.

The current 8-item biomarker panel was identified in a subset of subjects of the Maastricht IBS cohort. Although external validation in a second independent clinical IBS cohort is needed, we validated the biomarker panel in the pre-assigned independent validation set of IBS patients and controls of the Maastricht IBS cohort. These subjects were not used in the biomarker panel discovery step. The LifeLines DEEP cohort was a large population based cohort in which biosamples and symptom scores were collected. This cohort was not included in the current study as an external validation cohort, but to test the potency of the biomarker panel to pick up the presence of gastro-intestinal symptoms in the general population.

Symptom reduction is the primary goal of therapeutic interventions in IBS. Symptom assessment, mostly by retrospective paper surveys, is challenging and is subject to recall bias and fake compliance^47^. Intervention trials in IBS patients using symptom scores as main outcomes are hampered by high placebo response^48^. The identified biomarker panel was found to correlate moderately though significantly (*r* = 0.59, *p* < 0.001) with GI symptom severity in the IBS cohort. This indicates a possible potential of the biomarker panel to quantify responsiveness to treatments in addition to symptom scores, in clinical trials. However, this hypothesis needs to be tested further.

IBS is a highly prevalent disorder among the general population, but difficult to identify in subjects not seeking medical attention. Furthermore, a large part of subjects with IBS symptoms does not seek medical attention but try to improve their well-being by self-treatment. The evaluation of potential beneficial effects of interventions such as food products, supplementals or life style factors on GI health in the general population is challenging, since at group level GI symptom severity is low. Alternatively, a biomarker panel next to GI symptom questionnaires may have added value to quantify improvements by a specific intervention in the general population. Since our biomarker set is based on multiple gut health domains and correlated significantly with symptoms in IBS patients, we hypothesized that it would also be applicable in the general population. As expected, the majority of subjects form the general population reported to have no or hardly any GI symptoms, resulting in low overall mean symptom scores. As proof of principle, subjects with a 7-day mean score of 1.5 or higher for abdominal pain and discomfort were selected. In this study subgroup, a significant moderate correlation (*r* = 0.51, *p* = 0.003) between the 8-item biomarker panel and GI symptom scores was observed. This may indicate a potential application of the current non-invasive 8-item biomarker panel in research focusing on gut health and possible interventions thereof in the general population. Furthermore, as the biomarker panel reflects various domains of gut health and distinguishes patients with a functional GI disorder from healthy subjects, it may be a first step in the development of a biomarker (set) for healthy gut function.

## Conclusion

A novel, non-invasive, ‘multi-gut health domain’ biomarker panel, with a high predictive value for the discrimination between IBS patients and healthy controls, has been identified and validated. Furthermore, a moderate and highly significant correlation was observed between the biomarker panel and GI symptom severity in a clinical IBS cohort as well as in a general population cohort.

## Additional Information

**How to cite this article**: Mujagic, Z. *et al*. A novel biomarker panel for irritable bowel syndrome and the application in the general population. *Sci. Rep*. **6**, 26420; doi: 10.1038/srep26420 (2016).

## Figures and Tables

**Table 1:** Baseline characteristics of the MIBS cohort groups (*i.e*. IBS patients versus HC).

Parameter	IBS (n = 196)	HC (n = 160)
Demographics and Lifestyle		
Age (mean years ± SD)	44.8 ± 16.4	43.9 ± 19.2
Female sex (%)	70.4	61.3
BMI (mean kg/m^2^ ± SD)	25.0 ± 4.6*	24.0 ± 3.9
Duration of IBS symptoms (mean years ± SD)	13.6 ± 10.8	–
IBS subtype: IBS-D/IBS-C/IBS-M/IBS-U (n)	71/34/78/13	–
Current or previous smoker (%)	51.6***	39.3
Alcohol abstainers: 0 units/week (%)	42.1***	19.1
Moderate alcohol use: 1–15 units/week (%)	54.7**	70.7
GI symptoms assessed by end-of-day diary, 14-day mean ± SD		
Abdominal pain	2.3 ± 0.9***	1.1 ± 0.2
Abdominal discomfort	2.4 ± 0.8***	1.1 ± 0.2
Bloating	2.2 ± 0.9***	1.1 ± 0.2
Belching	1.6 ± 0.7***	1.1 ± 0.3
Nausea	1.6 ± 0.8***	1.0 ± 0.1
Flatulence	2.3 ± 0.9***	1.3 ± 0.5
Constipation	1.5 ± 0.7***	1.1 ± 0.2
Diarrhea	1.5 ± 0.6***	1.0 ± 0.1

Differences tested with independent samples t-test and Pearson Chi^2^; *p < 0.05; **p < 0.01; ***p < 0.001 vs. HC.

**Table 2:** Levels of biomarkers of the MIBS cohort (*i.e*. IBS patients versus HC).

Plasma biomarkers (median [Q1; Q3])	IBS (n = 196)	HC (n = 160)	*p*-value
Citrulline (μmol/l)	41.9 [33.9; 50.5]	39.3 [33.0; 47.2]	NS
IL-1β (μg/l)	0.15 [0.08; 0.71]	0.19 [0.10; 1.46]	0.01
IL-6 (μg/l)	0.24 [0.12; 0.36]	0.52 [0.20; 3.13]	<0.001
IL-8 (μg/l)	1.69 [0.95; 2.52]	1.60 [1.14; 2.45]	NS
IL-10 (μg/l)	0.86 [0.69; 1.09]	0.84 [0.61; 1.04]	NS
IL-12p70 (μg/l)	2.19 [1.21; 2.99]	1.20 [0.11; 2.00]	<0.001
TNF-α (μg/l)	0.14 [0.07; 3.20]	0.13 [0.06; 1.53]	NS
Fecal biomarkers (median [Q1; Q3])			
Chromogranin A (CgA) (nmol/g)	15.2 [7.6; 45.8]	9.4 [6.3; 27.3]	0.001
Calprotectin (μg/g)	37.3 [18.7; 73.5]	21.6 [6.8; 47.8]	<0.001
Human *β*-defensin 2 (HBD2) (ng/g)	31.3 [18.8; 51.9]	38.4 [27.1; 60.5]	<0.01
SCFA (C2): Acetate (μmol/g)	33.4 [22.5; 47.1]	35.7 [25.9; 47.4]	NS
SCFA (C3): Propionate (μmol/g)	9.5 [6.8; 14.0]	9.5 [6.6; 13.8]	NS
SCFA (C4): Butyrate (μmol/g)	7.8 [4.6; 13.2]	9.4 [5.9; 13.5]	NS
SCFA (C5): Valerate (μmol/g)	1.2 [0.8; 1.8]	1.5 [1.0; 2.1]	0.01
SCFA (C6): Caproate (μmol/g)	0.1 [0.0; 0.5]	0.3 [0.1; 0.9]	<0.001

Difference tested with Mann Whitney U test (presented *p*-value). Statistically significant differences did not diminish after post-hoc correction for multiple testing by Benjamini–Hochberg step up procedure.

**Table 3:** Baseline characteristics of the LL DEEP cohort: general population.

Parameters of general population subjects	n = 958
Demographics and Lifestyle	
Age (mean years ± SD)	45.2 ± 13.2
Female sex (%)	58.5
BMI (mean kg/m^2^ ± SD)	25.5 ± 4.5
Current smokers (%)	18.9
GI symptoms assessed by end-of-day diary, 7-day mean ± SD	
Abdominal pain	1.2 ± 0.4
Abdominal discomfort	1.3 ± 0.5
Bloating	1.4 ± 0.5
Belching	1.1 ± 0.3
Nausea	1.1 ± 0.3
Flatulence	1.6 ± 0.6
Constipation	1.1 ± 0.4
Diarrhea	1.1 ± 0.3

**Table 4:** Levels of biomarkers of the LL DEEP cohort: general population.

Plasma biomarkers (median [Q1; Q3])	n = 958
Citrulline (μmol/l)	41.2 [34.7; 49.1]
IL-1β (μg/l)	0.11 [0.10; 1.00]
IL-6 (μg/l)	0.19 [0.18; 0.20]
IL-8 (μg/l)	1.91 [1.30; 2.77]
IL-10 (μg/l)	0.89 [0.56; 1.23]
IL-12p70 (μg/l)	1.14 [0.06; 2.25]
TNF-α (μg/l)	0.08 [0.07; 0.09]
Fecal biomarkers (median [Q1; Q3])	
Chromogranin A (CgA) (nmol/g)	11.1 [7.2; 23.4]
Calprotectin (μg/g)	31.6 [10.5; 60.4]
Human β-defensin 2 (HBD2) (ng/g)	36.8 [22.5; 62.2]
SCFA (C2): Acetate (μmol/g)	27.8 [19.6; 47.1]
SCFA (C3): Propionate (μmol/g)	7.8 [5.2; 11.6]
SCFA (C4): Butyrate (μmol/g)	7.9 [5.0; 12.5]
SCFA (C5): Valerate (μmol/g)	1.4 [1.0; 2.0]
SCFA (C6): Caproate (μmol/g)	0.3 [0.1; 0.8]
